# A Clinically Aligned Two‐Stage Machine Learning Framework for Predicting Hungry Bone Syndrome After Parathyroidectomy

**DOI:** 10.1002/edm2.70297

**Published:** 2026-07-30

**Authors:** Shih‐Min Yin, Yu‐Chieh Lin, Tzu‐Hsun Hung, Shen‐En Chou, Shun‐Yu Chi, Fong‐Fu Chou, Yi‐Ju Wu, Si‐Yuan Wu, Jenn‐Jier James Lien, Po‐Chih Kuo, Yi‐Chia Chan

**Affiliations:** ^1^ Department of General Surgery Kaohsiung Chang Gung Memorial Hospital Kaohsiung Taiwan; ^2^ Department of Computer Science and Information Engineering National Cheng Kung University Tainan Taiwan; ^3^ Department of Computer Science National Tsing Hua University Hsinchu Taiwan; ^4^ Institute of Electrical Engineering, National Tsing Hua University Hsinchu Taiwan; ^5^ Division of General Surgery, Department of Surgery Tri‐Service General Hospital, National Defense Medical University Taipei Taiwan; ^6^ Kaohsiung Municipal Fong Shan Hospital of Chang Gung Medical Foundation Kaohsiung Taiwan

**Keywords:** hungry bone syndrome, machine learning, parathyroidectomy, risk prediction, secondary hyperparathyroidism

## Abstract

**Background:**

Hungry bone syndrome (HBS) is a frequent and clinically significant complication following parathyroidectomy (PTX) in patients with secondary hyperparathyroidism (SHPT), often leading to prolonged hypocalcaemia and increased healthcare burden. Existing prediction models are limited by small sample sizes and inability to capture complex clinical interactions. This study aimed to develop and validate a clinically aligned, two‐stage machine learning (ML) framework to predict HBS after PTX.

**Materials and Methods:**

A retrospective cohort of patients undergoing PTX for SHPT between 2008 and 2025 at a tertiary centre was analysed. A two‐stage ML framework was constructed: stage 1 used preoperative variables to generate a risk score, and stage 2 integrated this score with intraoperative features. Multiple ML models were evaluated using area under the receiver operating characteristic curve (AUROC), calibration metrics and resampling techniques.

**Results:**

A total of 882 patients were included, with an HBS incidence of 69.9%. EasyEnsemble and logistic regression demonstrated the highest discrimination (AUROC 0.712), outperforming the *k*‐nearest neighbours baseline. EasyEnsemble achieved the best overall performance (accuracy 0.707, F1 score 0.666) and calibration (Brier score 0.186). Key predictors included elevated preoperative alkaline phosphatase, higher intact parathyroid hormone levels and lower serum calcium.

**Conclusion:**

This two‐stage ML framework demonstrated acceptable predictive performance and aligns with clinical decision‐making processes. It enables early identification of high‐risk patients and may support individualised perioperative management to mitigate HBS and its complications.

## Graphical abstract

A clinically aligned two‐stage machine learning framework integrates preoperative and perioperative features to predict hungry bone syndrome after parathyroidectomy. Explainable risk stratification may support individualised calcium management and early intervention for high‐risk patients.

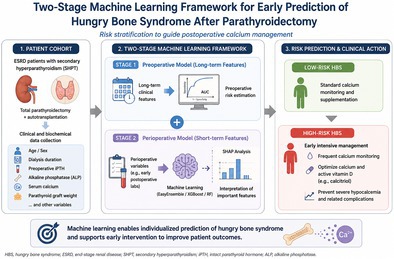

**Abbreviations**
ALP: alkaline phosphatase
EBL: estimated blood loss
ESRD: end‐stage renal disease
HBS: hungry bone syndrome
HPT: hyperparathyroidism
iPTH: intact parathyroid hormone
ML: machine learning
PTX: parathyroidectomy
SHPT: secondary hyperparathyroidism

## 1 Introduction

Secondary hyperparathyroidism (SHPT) remains highly prevalent among patients with end‐stage renal disease (ESRD) receiving maintenance dialysis. When biochemical control is suboptimal, SHPT is associated with clinically significant complications, including high‐turnover renal osteodystrophy with bone pain and fragility fractures, and extra skeletal calcification, such as calcific uremic arteriolopathy, with a concomitant increase in cardiovascular morbidity [1, 2, 3]. Although contemporary medical therapy has expanded to include vitamin D analogues, phosphate binders and calcimimetics, a substantial proportion of patients receiving dialysis still develop refractory SHPT. Across cohorts, approximately 15%–40% ultimately require parathyroidectomy (PTX), which is generally regarded as the definitive treatment for medically resistant disease [4, 5]. PTX is associated with improved survival, reduced cardiovascular events, and better control of hypercalcemia, hyperphosphatemia and bone mineral density, especially in patients severely refractory to medical therapy [6, 7, 8].

Hungry bone syndrome (HBS) is one of the most common and significant postoperative complications in patients with SHPT undergoing PTX. The incidence of HBS ranges from approximately 19% to 76%, depending on the population, severity of the disease and different surgical approaches [9, 10, 11, 12]. Prolonged hypocalcaemia caused by HBS can result in neuromuscular manifestations, including paraesthesia, muscle cramps and seizures or laryngospasm in severe cases. Cardiac complications, including QT prolongation and arrhythmias, may occur and can be life‐threatening [13, 14, 15]. Patients with severe HBS may require intensive intravenous calcium and active vitamin D supplementation, which can prolong hospitalisation. The correction of hypocalcaemia and the associated hypophosphatemia and hypomagnesemia may even continue for weeks to months to correct. Therefore, timely risk stratification for HBS and early therapeutic intervention is essential to optimise postoperative management and improve clinical outcomes.

Numerous recent studies consistently identify high preoperative bone turnover and disease severity markers as the strongest predictors of HBS after PTX for SHPT patients, including markedly elevated preoperative intact parathyroid hormone (iPTH) levels and alkaline phosphatase (ALP) [9, 16]. In addition, patients who develop severe hypocalcaemia or absence of hypercalcemia are significantly more prone to HBS [9, 17]. Other studies also identified younger age, prolonged dialysis period, absence of autotransplantation and increased total resected parathyroid gland weight as consistently associated with HBS severity [9, 17, 18, 19]. Despite these established associations, existing risk scores and nomograms are predominantly derived from small, single‐centre cohorts lacking external validation, which limits their generalisability. Additionally, traditional linear statistical models often fail to capture the complex, non‐linear interactions between these multifaceted variables.

The present study aims to develop and validate a machine learning (ML) based workflow to predict the occurrence of HBS using preoperative patient characteristics and intraoperative findings. In addition, we aim to identify early surrogate risk factors for subsequent HBS after PTX in patients with SHPT.

## 2 Method

### 2.1 Patient Demographics and Variable Collection

This retrospective study included patients who underwent PTX for HPT at a single tertiary centre from January 2008 through March 2025. The study was conducted in accordance with the Declaration of Helsinki and was approved by the Institutional Review Board of Chang Gung Medical Foundation (IRB No. 202600215B0). The requirement for informed consent was waived due to the retrospective study design.

Patients with primary or tertiary HPT, those undergoing reoperation for recurrent disease, and those without complete perioperative serum calcium or iPTH data were excluded. Clinical variables were abstracted from electronic medical records and included demographics, comorbidities, dialysis characteristics, presenting symptoms, operative procedures, preoperative laboratory values and intraoperative findings. Operative duration, estimated blood loss and resected gland size and weight were recorded, and cumulative gland weight was calculated for each patient. Postoperative serum calcium and iPTH levels were monitored, and HBS was defined as serum total calcium level less than 8.4 mg/dL persisting for more than four consecutive postoperative days [14].

### 2.2 Perioperative Clinical Protocol for PTX Patients

All PTX procedures were performed by 1 of 5 senior endocrine surgeons using a standardised operative approach. Surgeons systematically explored the neck to identify four parathyroid glands and performed complete excision, and the smallest gland was selected for autotransplantation into the patient's forearm without an arteriovenous fistula, typically with 100 or 140 mg of parathyroid tissue. When fewer than four glands could be identified intraoperatively, surgeons were permitted to proceed with PTX without autotransplantation. Concomitant thyroidectomy was performed only when a separate surgical indication for thyroid disease was present. After surgery, all patients received prophylactic oral calcium carbonate (500 mg, 3 times daily) and active vitamin D (calcitriol, 0.25 μg, twice daily) 1 day after surgery. Serum calcium and iPTH levels were routinely assessed on postoperative days 1 and 3, and supplementation was titrated according to symptoms and measured levels; intravenous calcium gluconate was administered when clinically indicated for symptomatic or severe hypocalcaemia, in accordance with protocols described in previous studies [20].

### 2.3 Data Preprocessing

Data preprocessing was performed before model development to optimise data quality and minimise bias. The dataset was randomly partitioned into training and testing cohorts in an 8:2 ratio, and this split was maintained across all modelling procedures. To prevent information leakage, all preprocessing parameters were fitted only on the training set and then applied to the test set. Variables with more than 15% missingness were excluded in the training set using a prespecified data‐quality threshold based on clinician‐guided review of missing‐data patterns and imputation reliability (Table S1), and the same feature set was then applied to the independent test set. For remaining variables, missing values were imputed using an iterative approach that compared multiple base estimators, including *k*‐nearest neighbours (kNN), random forest (RF), support vector regression (SVM) and gradient boosting regressors (XGBoost). During cross‐validation, imputation models were selected and fitted only within the corresponding training fold. Imputation adequacy was additionally evaluated by comparing the root mean squared error with the observed standard deviation for each variable. Continuous variables were standardised as *z* scores, and categorical variables were one‐hot encoded.

### 2.4 Two‐Stage Machine Learning Framework

To estimate the risk of postoperative HBS, we constructed a two‐stage ML framework designed to mirror the temporal sequence of surgical decision making. Long‐term features comprised preoperative demographics, comorbidities, dialysis history and routine laboratory tests. During cross‐validation, the 14 features were reselected within each training fold. Short‐term features comprised intraoperative parameters, including surgical approach, operative duration, estimated blood loss (EBL), total parathyroid gland size and weight and concomitant procedures.

In stage 1, an XGBoost model was trained using preoperative long‐term features to generate an individualised preoperative risk score, defined as the predicted probability of HBS. Internal validation was performed within the training set using repeated stratified fivefold cross‐validation, repeated 10 times to yield 50 validation iterations. In each iteration, the complete two‐stage pipeline was retrained. To avoid optimistic stacking, stage 1 risk scores used for stage 2 training were generated as out‐of‐fold predictions within the training data.

**FIGURE 1:**
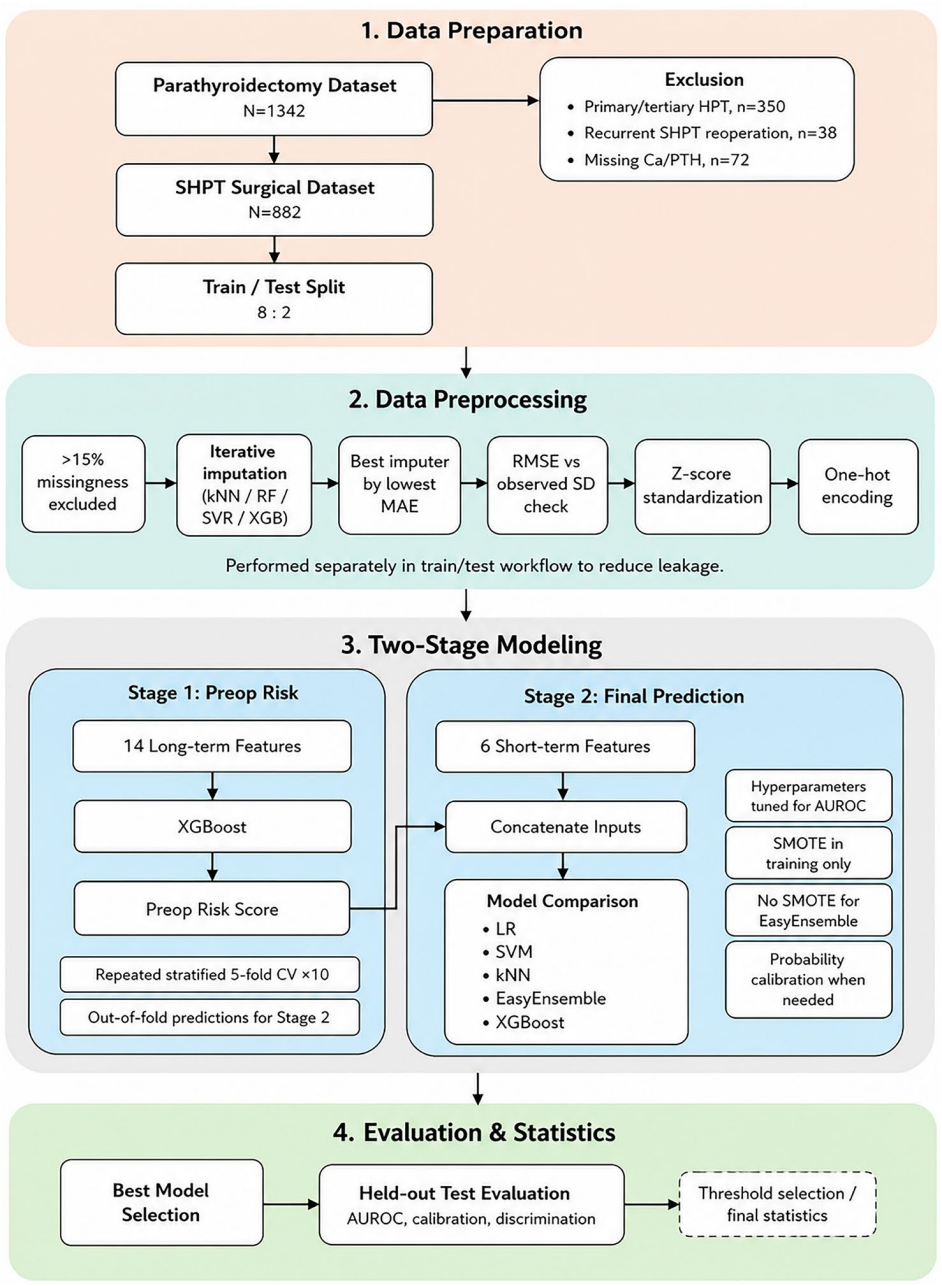
Two‐stage machine learning framework for predicting hungry bone syndrome after parathyroidectomy. Overview of the two‐stage workflow in which an XGBoost‐based stage 1 model generates an individualised preoperative risk score from long‐term features, which is then combined with prespecified intraoperative features for stage 2 model training and comparison across classifiers.

In stage 2, the stage 1 risk score was concatenated with six prespecified short‐term features and used as the unified input for final model comparison. We evaluated logistic regression, SVM, kNN, EasyEnsemble and XGBoost, with hyperparameters tuned to maximise the receiver operating characteristic curve (AUROC). SMOTE was applied only within the training fold to address class imbalance. EasyEnsemble was not combined with SMOTE because it intrinsically manages imbalanced classification. Probability calibration was performed within the training workflow when required. The detailed ML framework was demonstrated in Figure 1.

### 2.5 Model Evaluation and Statistics Analysis

Model discrimination was quantified using the area under the receiver operating characteristic curve with percentile bootstrap confidence intervals. For distinction of model‐selection domain, calibration was assessed visually with calibration curves and summarised using the Brier score and expected calibration error (ECE). Classification performance was demonstrated by sensitivity (recall), specificity, accuracy, precision and F1 score at the Youden index‐derived cut‐off. Since the model was intended for screening‐oriented risk stratification of HBS, final model selection was based on integrated assessment of discrimination, calibration and sensitivity‐first classification ability. Feature importance was further assessed using SHAP to identify the major predictors contributing to HBS risk estimation.

Continuous variables are presented as mean (standard deviation) or median (interquartile range), as appropriate, with normality assessed using the Shapiro–Wilk test. Between‐group comparisons used the Student's *t*‐test for normally distributed variables and the Mann–Whitney *U* test for non‐normally distributed variables. Categorical variables are reported as counts (percentages) and compared using the Pearson chi‐square test. Risk factors were evaluated using univariate and multivariate logistic regression analyses. Statistical significance was defined as a 2‐sided *α* of 0.05. Analyses were performed using Stata, version 16.1 and Python, version 3.10.4, with model training, resampling and visualisation implemented using standard scientific computing libraries.

## 3 Result

### 3.1 Patient Demographics and Baseline Characteristics of Variables

Between January 2008 and March 2025, 1342 patients who underwent PTX were identified. A total of 350 procedures for primary or tertiary HPT, 38 reoperations for recurrent SHPT and 72 SHPT cases with missing perioperative Ca and iPTH data were excluded (Figure 1). The baseline characteristics of variables by HBS status were demonstrated in Table 1. The overall incidence of HBS was 69.9% in this study. Patients who developed HBS were younger and more often reported itching. The distribution of OP method also differed, with a higher proportion receiving total PTX with autotransplantation of 140 g, and a higher proportion of no concomitant thyroidectomy. Preoperative iPTH and ALP levels were higher in the HBS group, whereas serum calcium level was lower. Additionally, higher serum phosphate and serum calcium × phosphate product were also observed in the HBS group. Table 2 summarises the descriptive statistics for all variables in the preprocessed training/validation and test cohorts. No significant differences between the datasets were observed.

**TABLE 1:** Baseline characteristics of variables by hungry bone syndrome status.

Variable	No HBS	HBS present	*p*	SMD	Adjusted *p* value (BH)
	*n* = 265	*n* = 617			
Sex, *n* (%)			0.607	0.043	0.686
Male	112 (42.3)	274 (44.4)			
Female	153 (57.7)	343 (55.6)			
Age (years), mean (SD)	58.8 ± 10.7	54.5 ± 12.1	< 0.001	0.374	0.003
BMI (kg/m^2^), mean (SD)	23.0 ± 3.7	23.5 ± 3.9	0.056	0.139	0.120
Clinical symptoms, *n* (%)					
Bone pain	145 (54.7)	357 (57.9)	0.429	0.063	0.531
Calciphylaxis	43 (16.2)	70 (11.3)	0.060	0.142	0.120
Itching	119 (44.9)	325 (52.7)	0.041	0.156	0.097
Weakness	85 (32.1)	199 (32.3)	1.000	0.004	1.000
ESRD years (years)	9.2 ± 5.2	8.8 ± 4.9	0.288	0.079	0.394
Dialysis modality, *n* (%)			0.066	0.156	0.123
HD	236 (89.1)	552 (89.5)			
CAPD	21 (7.9)	59 (9.6)			
CAPD first, then shift to HD	8 (3.0)	6 (0.9)			
Diabetes, *n* (%)	58 (21.9)	104 (16.9)	0.094	0.128	0.163
Hypertension, *n* (%)	183 (69.1)	405 (65.6)	0.363	0.073	0.472
OP time (min), mean (SD)	150.0 ± 47.8	146.1 ± 47.0	0.264	0.082	0.381
OP method, *n* (%)			< 0.001	0.337	0.003
tPTX + AT 100 g	104 (39.2)	178 (28.8)			
tPTX + AT 140 g	121 (45.7)	375 (60.8)			
tPTX only	40 (15.1)	61 (9.9)			
Subtotal PTX	0 (0)	3 (0.5)			
EBL (mL), mean (SD)	17.0 ± 11.9	18.3 ± 16.9	0.173	0.093	0.265
Total gland size (cm), mean (SD)	6.2 ± 1.4	6.6 ± 1.8	0.002	0.221	0.006
Total gland weight (g), mean (SD)	4.0 ± 5.5	4.1 ± 2.7	0.800	0.021	0.867
Thymectomy, *n* (%)	196 (74.0)	459 (74.4)	0.960	0.010	0.998
Thyroidectomy status, *n* (%)			< 0.001	0.315	0.003
No thyroidectomy	194 (73.2)	525 (85.1)			
Left lobectomy	11 (4.2)	17 (2.8)			
Right lobectomy	16 (6.0)	15 (2.4)			
Bilateral total thyroidectomy	39 (14.7)	48 (7.8)			
Partial excision of thyroid	5 (1.9)	12 (1.9)			
PTH (pg/mL), mean (SD)	1351.0 ± 880.9	1742.1 ± 1221.3	< 0.001	0.367	0.003
Hb (g/dL), mean (SD)	11.25 ± 1.47	11.10 ± 1.76	0.155	0.101	0.252
ALP, mean (SD)	161.1 ± 110.0	255.8 ± 234.3	< 0.001	0.518	0.003
Ca, mean (SD)	10.64 ± 0.99	10.29 ± 0.86	< 0.001	0.377	0.003
P, mean (SD)	5.40 ± 1.51	6.00 ± 2.19	< 0.001	0.322	0.003
Ca × P, mean (SD)	57.07 ± 15.47	61.70 ± 23.95	< 0.001	0.229	0.003
PTH/ESRD years, mean (SD)	470.2 ± 1150.8	309.9 ± 478.1	0.029	0.182	0.075
OP time × EBL, mean (SD)	2686.9 ± 2462.9	2829.8 ± 3656.9	0.499	0.046	0.590

*Note:* False discovery rate (FDR) correction using the Benjamini–Hochberg procedure was applied. Adjusted *p* values are reported where appropriate.

Abbreviations: ALK, alkaline phosphatase; AT, autotransplantation; BMI, body mass index; Ca, calcium; CAPD, continuous ambulatory peritoneal dialysis; EBL, estimated blood loss; ESRD, end‐stage renal disease; Hb, haemoglobin; HBS, hungry bone syndrome; HD, haemodialysis; P, phosphate; PTH, parathyroid hormone; SD, standard deviation; SMD, standardised mean difference (computed from individual‐level data; absolute values reported); tPTX, total parathyroidectomy.

**TABLE 2:** Baseline characteristics between training, validation and testing dataset.

Variable	Training/validation	Testing	*p*	SMD
	*n* = 705	*n* = 177		
HBS, *n* (%)	493 (69.9)	124 (70.1)		0.003
Sex, *n* (%)			0.233	0.107
Male	301 (42.7)	85 (48.0)		
Female	404 (57.3)	92 (52.0)		
Age (years), mean (SD)	60.0 ± 11.7	55.1 ± 12.5	0.374	0.079
BMI (kg/m^2^), mean (SD)	23.4 ± 3.8	23.5 ± 4.0	0.567	0.049
Clinical symptoms, *n* (%)				
Bone pain	408 (57.9)	94 (53.1)	0.289	0.096
Calciphylaxis	93 (13.2)	20 (11.3)	0.584	0.058
Itching	359 (50.9)	85 (48.0)	0.545	0.058
Weakness	228 (32.3)	56 (31.6)	0.929	0.015
ESRD years (years)	8.9 ± 4.9	9.0 ± 5.3	0.928	0.008
Dialysis modality, *n* (%)			0.947	0.028
HD	583 (88.9)	159 (89.8)		
CAPD	65 (9.2)	15 (8.5)		
CAPD first, then shift to HD	11 (1.6)	3 (1.7)		
Diabetes, *n* (%)	131 (18.6)	31 (17.5)	0.826	0.028
Hypertension, *n* (%)	235 (66.7)	118 (66.7)	1.000	0.000
OP time (min), mean (SD)	147.3 ± 47.1	147.0 ± 48.2	0.939	0.007
OP Method, *n* (%)			0.664	0.102
tPTX + AT 100 g	225 (31.9)	56 (32.8)		
tPTX + AT 140 g	401 (56.9)	94 (53.1)		
tPTX only	77 (10.9)	24 (13.6)		
Three and half PTX	2 (0.3)	1 (0.5)		
EBL (mL), mean (SD)	18.11 ± 16.34	17.15 ± 11.91	0.376	0.067
Total gland size (cm), mean (SD)	6.4 ± 1.7	6.5 ± 1.8	0.614	0.043
Total gland weight (g), mean (SD)	4.1 ± 4.0	4.0 ± 2.8	0.695	0.029
Thymectomy, *n* (%)	527 (74.8)	128 (72.3)	0.571	0.055
Thyroidectomy status, *n* (%)			0.918	0.084
No thyroidectomy	571 (81.0)	148 (83.6)		
Left lobectomy	22 (3.1)	6 (3.4)		
Right lobectomy	26 (3.7)	5 (2.8)		
Bilateral total thyroidectomy	72 (10.2)	15 (8.5)		
Partial excision of thyroid	14 (2.0)	3 (1.7)		
PTH (pg/mL), mean (SD)	1617.1 ± 1119.1	1654.3 ± 1239.0	0.717	0.031
Hb (g/dL), mean (SD)	11.15 ± 1.69	11.06 ± 1.62	0.500	0.056
ALK, mean (SD)	223.9 ± 212.1	241.2 ± 198.9	0.309	0.084
Ca, mean (SD)	10.40 ± 0.95	10.38 ± 0.76	0.794	0.020
P, mean (SD)	5.76 ± 1.47	6.06 ± 3.44	0.258	0.113
Ca × P, mean (SD)	59.64 ± 14.78	62.98 ± 38.82	0.262	0.114
PTH/ESRD years, mean (SD)	347.3 ± 715.0	400.9 ± 875.4	0.452	0.067
OP time × EBL, mean (SD)	2826.2 ± 3557.5	2630.2 ± 2295.6	0.370	0.065

Abbreviations: ALK, alkaline phosphatase; AT, autotransplantation; BMI, body mass index; Ca, calcium; CAPD, continuous ambulatory peritoneal dialysis; EBL, estimated blood loss; ESRD, end‐stage renal disease; Hb, haemoglobin; HBS, hungry bone syndrome; HD, haemodialysis; P, phosphate; PTH, parathyroid hormone; SD, standard deviation; SMD, standardised mean difference (computed from individual‐level data; absolute values reported); tPTX, total parathyroidectomy.

### 3.2 Machine Learning Based Prediction of HBS

**FIGURE 2:**
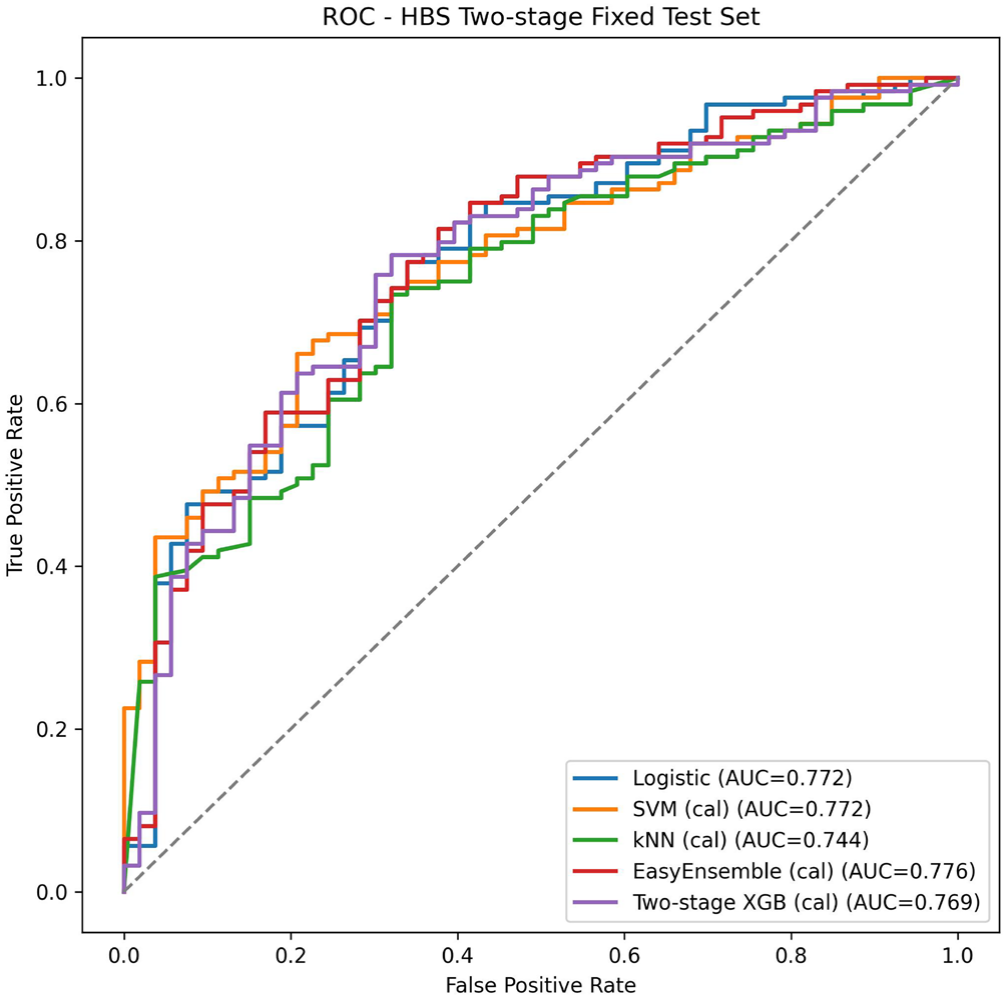
AUROC of machine learning models for predicting hungry bone syndrome. The receiver operating characteristics curve and area under the receiver operating characteristic (AUROC) curve for the evaluated classifiers in the independent testing dataset.

**FIGURE 3:**
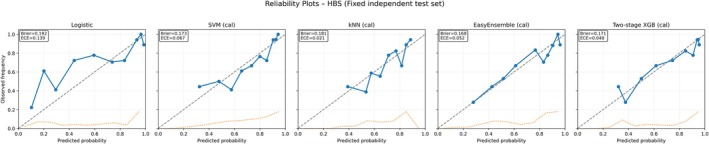
Model calibration and feature attribution for hungry bone syndrome prediction. Calibration performance of the evaluated models summarised by calibration curves and Brier score/ECE.

Following the two‐stage model training, five ML classifiers against the independent testing dataset were compared based on discrimination, calibration and classification performance. As shown in Figure 2 and Table 3, EasyEnsemble achieved the highest AUROC of 0.776, the lowest Brier score of 0.168, and a low ECE of 0.052. Logistic regression had a similar AUROC of 0.772 but poorer calibration, with a Brier score of 0.192 and ECE of 0.139. Two‐stage XGBoost achieved a slightly lower AUROC and higher Brier score than EasyEnsemble despite a lower ECE of 0.048. kNN had the lowest ECE of 0.021 but the lowest AUROC of 0.744 and a higher Brier score of 0.181 (Figure 3).

**TABLE 3:** Model performance comparison for HBS.

Model	AUROC	Brier	ECE	Cut‐off	*p*
EasyEnsemble	0.776	0.168	0.052	0.575	0.307
SVM	0.772	0.173	0.067	0.744	0.186
Logistic	0.772	0.192	0.139	0.454	0.367
Two‐stage XGB	0.769	0.171	0.048	0.613	0.382
kNN	0.744	0.181	0.021	0.669	Reference

For classification performance, EasyEnsemble achieved an AUROC of 0.776, with the highest recall of 0.815, accuracy of 0.757 and F1‐score of 0.824 among all evaluated models. Two‐stage XGB demonstrated identical performance, each with a recall of 0.782, accuracy of 0.751 and F1 score of 0.815. While the SVM showed the lowest recall of 0.661, accuracy of 0.701 and F1 score of 0.756 compared to other models (Table 4).

**TABLE 4:** Performance metrics of different models for HBS.

Model	Accuracy	Precision	Sensitivity (recall)	F1‐score	Specificity
EasyEnsemble	0.757	0.835	0.815	0.824	0.623
SVM	0.701	0.882	0.661	0.756	0.792
Logistic	0.740	0.842	0.774	0.807	0.660
Two‐stage XGB	0.751	0.851	0.782	0.815	0.679
kNN	0.718	0.843	0.734	0.784	0.679

In ablation study, the EasyEnsmble model was also compared to the best model under different preprocessing settings, including single stage framework, model without SMOTE for class imbalance and model using preoperative features only. The results showed that although the no‐SMOTE and single‐stage models yielded slightly higher AUROC values of 0.786 and 0.782, respectively, these models demonstrated substantially lower recall than the two‐stage model. The results were demonstrated in Table S2.

Given the high incidence of HBS in this cohort and the clinical priority of minimising false‐negative predictions, the two‐stage EasyEnsemble was selected since it provided the most favourable integrated performance with highest AUROC, lowest Brier score, low ECE and better sensitivity‐oriented classification.

### 3.3 Features Importance of ML for HBS Prediction

**FIGURE 4:**
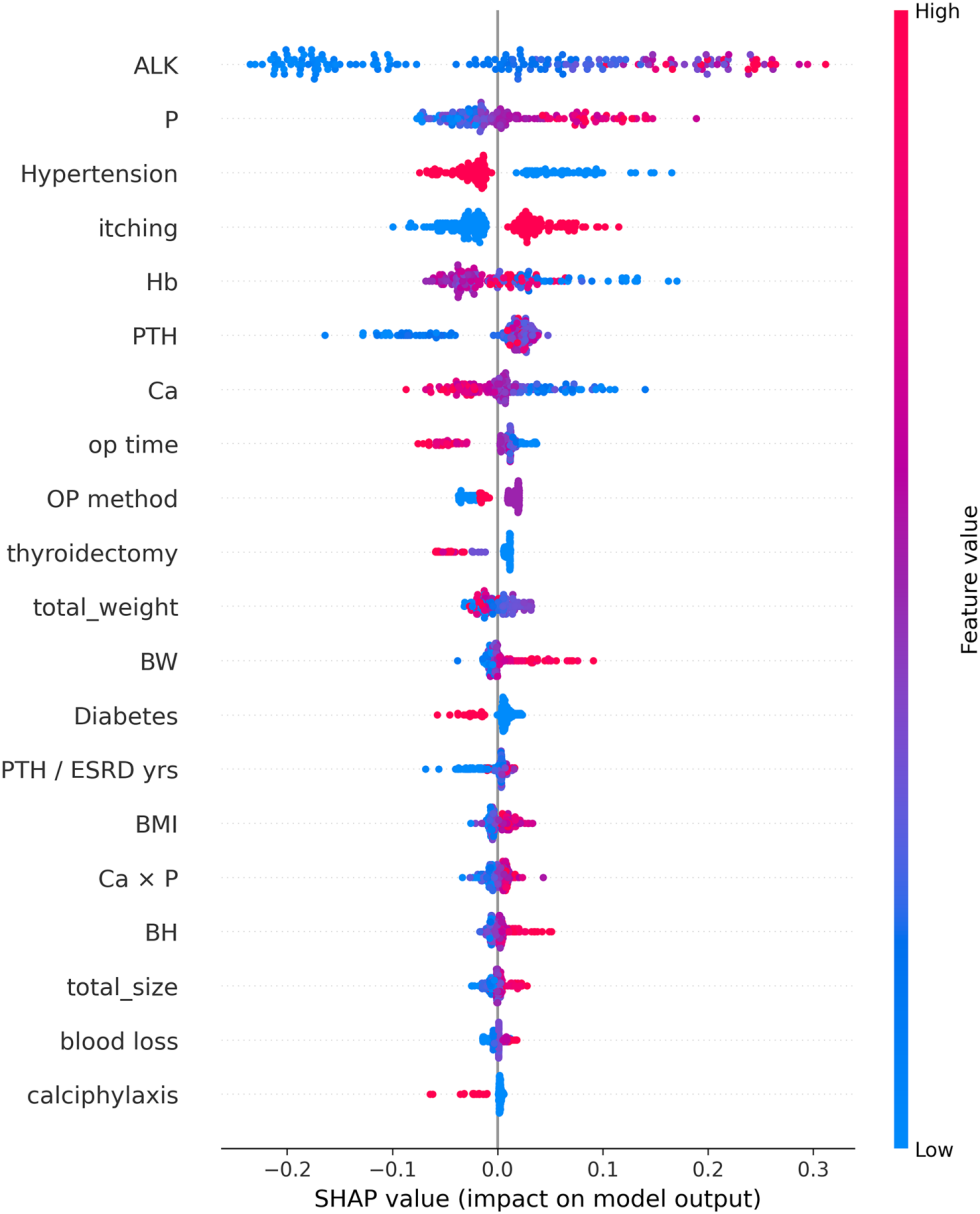
SHAP feature attribution for hungry bone syndrome prediction. SHAP summary plot quantifying the relative contribution of individual predictors to model output. ALK, alkaline phosphatase; AT, auto‐transplantation; BH, body height; BMI, Body mass index; BW, Body weight; Ca, serum calcium; EBL, Estimated blood loss; ESRD, end‐stage renal disease; Hb, haemoglobin; PTH, parathyroid hormone; P, serum phosphate; tPTX, total parathyroidectomy.

Permutation Shapley additive explanation analysis (SHAP) was performed to quantify the contribution of individual features to the prediction of HBS (Figure 4). To evaluate the independent contribution of each feature in two‐staged EasyEnsemble model, values across the dataset were systematically permuted and the resulting shifts in the model's predicted probabilities were measured. The importance of each feature was then directly quantified by the magnitude of the decline in overall predictive performance, demonstrating how critically the algorithm relies on that specific feature. Among all features, preoperative elevated ALP, phosphate, iPTH, decreased haemoglobin and calcium emerged as the strongest biochemical determinants of model output for predicting HBS. Furthermore, clinical manifestations such as pruritus, comorbidities including hypertension and intraoperative features including shorter operation time, PTX with 140 mg autotransplantation and absence of thyroidectomy were also significant determinants of model prediction. This result is compared to the multivariant regression analysis, which demonstrated that serum ALP, P, Ca, OP method and thyroidectomy status aligned with significant predictors for HBS (Table 5).

**TABLE 5:** Factors associated with postoperative hungry bone syndrome identified by univariable and multivariable logistic regression analyses.

Variable	Univariable odds ratio (95% CI)	*p*	Multivariable odds ratio (95% CI)	*p*
Laboratory variables				
ALP, per 10 U/L	1.05 (1.03–1.06)	< 0.001	1.04 (1.03–1.06)	< 0.001
Serum calcium, per 1 unit	0.64 (0.53–0.76)	< 0.001	0.64 (0.52–0.79)	< 0.001
Serum phosphorus, per 1 unit	1.29 (1.16–1.43)	< 0.001	1.21 (1.07–1.36)	0.002
PTH, per 100 pg/mL	1.06 (1.04–1.08)	< 0.001	1.02 (1.00–1.04)	0.081
Calcium × phosphorus, per 1 unit	1.02 (1.01–1.03)	< 0.001	Excluded (collinearity)	—
PTH/ESRD duration, per 100	0.98 (0.96–0.99)	0.007	0.97 (0.94–0.99)	0.015
Clinical characteristics				
Body weight, per 10 kg	1.16 (1.03–1.30)	0.015	1.18 (1.03–1.35)	0.015
Haemoglobin, per 1 unit	0.94 (0.87–1.03)	0.186	—	—
Itching	1.36 (1.02–1.82)	0.035	1.20 (0.87–1.66)	0.259
Calciphylaxis	0.66 (0.44–0.99)	0.046	1.01 (0.61–1.66)	0.977
Hypertension	0.86 (0.63–1.17)	0.331	—	—
BMI > 24 vs. ≤ 24	1.29 (0.96–1.74)	0.089	—	—
Diabetes	0.72 (0.50–1.03)	0.075	—	—
Combined thyroid surgery				
No thyroidectomy	Reference		Reference	
Left lobectomy	0.56 (0.26–1.22)	0.146	0.63 (0.26–1.52)	0.308
Right lobectomy	0.35 (0.17–0.72)	0.004	0.32 (0.14–0.74)	0.007
Bilateral total thyroidectomy	0.45 (0.29–0.72)	< 0.001	0.57 (0.35–0.94)	0.028
Partial thyroid excision	0.84 (0.30–2.39)	0.746	0.59 (0.19–1.83)	0.364
Surgical procedure				
tPTX + AT 100‐mg	Reference		Reference	
tPTX + AT 140‐mg	1.84 (1.34–2.53)	< 0.001	1.99 (1.38–2.87)	< 0.001
tPTX only	0.90 (0.56–1.43)	0.649	1.35 (0.79–2.32)	0.277
Subtotal PTX	4.14 (0.13–127.75)	0.417	6.20 (0.10–376.15)	0.384
Operative variables				
Operative time, per 60 min	0.90 (0.75–1.08)	0.257	—	—
Estimated blood loss, per 10 mL	1.06 (0.95–1.18)	0.286	—	—
Total gland weight, per unit	1.00 (0.96–1.04)	0.891	—	—
Total gland size, per unit	1.17 (1.05–1.30)	0.003	1.13 (1.00–1.27)	0.048
Thymectomy	1.03 (0.74–1.42)	0.881	—	—

Abbreviations: ALP, alkaline phosphatase; AT, autotransplantation; BMI, body mass index; CI, confidence interval; ESRD, end‐stage renal disease; HBS, hungry bone syndrome; OR, odds ratio; PTH, parathyroid hormone; PTX, parathyroidectomy; tPTX, total parathyroidectomy.

To facilitate clinical application, we further utilised the EasyEnsemble‐predicted probabilities to stratify patients into low‐, moderate‐ and high‐risk groups. A predicted probability of less than 35% was defined as low risk, 35%–85% as moderate risk and greater than 85% as high risk. The lower threshold of 35% was selected to prioritise sensitivity and minimise false‐negative classification, with the model achieving a sensitivity of 0.984 and negative predictive value (NPV) of 0.818. The upper threshold of 85% was selected to prioritise specificity and identify a high‐risk subgroup, with the model achieving a specificity of 0.849 and positive predictive value (PPV) of 0.886 (Table 6).

**TABLE 6:** Clinical risk stratification based on EasyEnsemble‐predicted probability of postoperative hungry bone syndrome.

Risk group	Predicted probability	*n* (%)	Observed HBS rate	Clinical interpretation
Low risk	< 35%	11/177 (6.2%)	18.2%	Rule‐out threshold: sensitivity 98.4%, NPV 81.8%
Moderate risk	35%–< 85%	96/177 (54.2%)	62.5%	Intermediate‐risk group between rule‐out and rule‐in thresholds
High risk	≥ 85%	70/177 (39.5%)	88.6%	Rule‐in threshold: specificity 84.9%, PPV 88.6%

*Note:* Observed HBS rates represent the proportion of patients with HBS within each predicted risk group.

Abbreviations: HBS, hungry bone syndrome; NPV, negative predictive value; PPV, positive predictive value.

## 4 Discussion

In this study, we developed an ML workflow that achieved acceptable performance for predicting HBS after PTX in SHPT patients using a large single‐centre cohort. The model was built on a structured two‐stage framework aligned with clinical workflow, initially estimating risk from preoperative features and subsequently refining predictions with intraoperative variables. Furthermore, the relative contribution of each variable was systematically assessed, with preoperative serum ALP, iPTH and calcium level emerging as the most significant features, as well as intraoperative factors such as total gland weight and operative time.

HBS with postoperative hypocalcaemia is the most frequent and clinically significant complication following PTX, which requires timely recognition and proactive management. Severe postoperative hypocalcaemia not only delays recovery but has also been associated with increased morbidity and healthcare utilisation [18, 21]. In a large population‐based analysis by Areef et al., emergency department visits or observation stays requiring hypocalcaemia treatment rose nearly 20‐fold compared with the preoperative year, underscoring the substantial clinical and economic burden of this complication [22]. Additionally, the incidence of HBS in our study from southern Taiwan reached 70%, similar to findings reported in a study from northern Taiwan [23]. In comparison with international reports, the Taiwanese population demonstrates a higher incidence of HBS, which may be attributable to the higher severity and burden of ESRD in Taiwan [24, 25].

Given the high incidence of HBS after PTX and its potential life‐threatening complications, numerous studies have aimed to estimate the predicting factors of HBS. Preoperative biochemical markers are the most consistently cited predictors across the studies, including high iPTH, elevated ALP and hypocalcaemia, reflecting a higher baseline bone‐remodelling status and an increased tendency for skeleton remineralisation after surgery [25, 26, 27, 28, 29]. Other clinical and anatomical determinants were also identified in different series, such as younger age, prolonged duration of dialysis, higher volume of the resected glands and lower pre‐operative haemoglobin and albumin [12, 28, 30]. However, most prior investigations of HBS risk factors have been limited by relatively small sample sizes and have focused predominantly on preoperative biochemical parameters, with limited incorporation of intraoperative features.

This study represents one of the largest single‐centre cohorts to date investigating HBS following PTX in patients with SHPT. Consistent with previous cohorts, this study confirmed that preoperative biochemical markers including serum iPTH, ALP and calcium level remain three of the most significant predictors for HBS. Notably, this study demonstrated the integration of intraoperative variables into the predictive framework, identifying additional total gland weight, operative time and different surgical approaches, which showed comparable predictive importance to established traditional risk factors such as hyperphosphatemia, dialysis duration and cumulative gland size. These findings were also aligned with previous research, which demonstrated larger parathyroid gland size has been associated with the occurrence of HBS, and subtotal PTX is associated with a lower incidence of HBS compared with PTX with autotransplantation [24, 25]. Additionally, the amount of transplanted parathyroid tissue may influence HBS occurrence. A 140‐mg autograft was associated with a higher incidence of HBS than a 100‐mg graft in both MI‐based framework and multivariate logistic regression analyses. Although graft neovascularisation may require 1 to 2 weeks after surgery, PTH secretion may begin earlier [31].

Based on our data, the incidence of HBS was lower in patients who underwent PTX combined with lobectomy or total thyroidectomy compared with those who underwent PTX alone. Although no clinical studies have directly supported this observation, bone metabolism is regulated not only by iPTH but also by thyroid hormones [32, 33]. Thyroid hormones stimulate osteoclast differentiation through the receptor activator of nuclear factor‐κB ligand (RANKL) pathway, thereby disrupting the balance between osteoclasts and osteoblasts [34, 35]. The altered interaction among iPTH, thyroid hormones and bone remodelling cells may contribute to dysregulation of calcium homeostasis, manifesting as HBS after concomitant thyroidectomy performed during PTX.

Two‐stage EasyEnsemble was selected as the final model in this study for the most clinically meaningful balance between discrimination, calibration and sensitivity‐oriented classification. For a perioperative HBS prediction tool, the primary objective is to minimise missed high‐risk cases that could benefit from closer postoperative surveillance and early calcium supplementation. False‐negative predictions may result in delayed recognition of patients at risk for HBS, potentially postponing intensified biochemical monitoring and preventive treatment. Therefore, model selection was not based solely on AUROC, but accurate identification of high‐risk patients and dependable risk prediction are both essential for guiding postoperative management and optimising patient safety.

To reduce complications of postoperative hypocalcaemia, current clinical guidelines and evidence support the routine use of prophylactic oral calcium supplementation without testing iPTH or calcium levels, as well as adding oral calcitriol to increase the effectiveness of oral calcium [36, 37, 38, 39]. However, evidence to guide medication adjustment among patients at high risk for HBS remains limited.

To facilitate clinical implementation, we translated the EasyEnsemble model into a three‐tier risk stratification system using two probability thresholds. Patients with a predicted HBS probability < 35% had a low observed HBS rate and may be suitable for standard postoperative management, whereas those with a predicted probability ≥ 85% had an observed HBS rate of 88.6% and may benefit from intensified serum calcium monitoring, earlier optimisation of calcium and active vitamin D supplementation, and closer postoperative surveillance. This risk‐stratified approach provides a practical framework for individualised postoperative management but requires external validation before routine clinical implementation.

This study had several limitations. First, the study did not capture long‐term hypocalcaemia status beyond the index postoperative period. Therefore, the model could only estimate short‐term risk of HBS rather than persistent hypocalcaemia, delayed recovery of parathyroid function or late readmissions. Second, although postoperative prophylactic calcium carbonate and vitamin D were routinely prescribed and intravenous calcium gluconate was administered as clinically indicated, complete time‐resolved medication records were unavailable, limiting adjustment for treatment intensity, dosing changes and adherence that could influence calcium kinetics and introduce residual confounding. Third, the study's retrospective design and long study period of 17 years may have introduced temporal confounding, as changes in dialysis management, surgical techniques and postoperative care may have occurred during the study period. Finally, although SHAP analysis identified the relative importance of individual predictors within the machine‐learning framework, it does not establish independent associations in the same manner as conventional multivariable regression models. Therefore, the identified feature rankings should be interpreted primarily as measures of ML model predictive contribution rather than independent effects. Further study is required to prospectively and externally validate this model in larger multicentre cohorts with more standardised operative definitions.

## 5 Conclusion

This study developed and validated a two‐stage ML framework that mirrors the temporal sequence of clinical decision making and demonstrated acceptable predictive performance with favourable calibration. Feature interpretability analyses identified clinically relevant preoperative and intraoperative predictors that contributed significantly to model output. This approach may facilitate earlier identification of high‐risk patients, enabling more intensive early postoperative serum calcium monitoring and timely escalation of calcium supplementation to mitigate progression to severe HBS.

## Author Contributions

**Yu‐Chieh Lin:** data curation, formal analysis, software. **Yi‐Chia Chan:** data curation, investigation, writing – review and editing. **Yi‐Ju Wu:** validation, supervision. **Jenn‐Jier James Lien:** formal analysis, writing – original draft. **Tzu‐Hsun Hung:** formal analysis, writing – original draft, software. **Shen‐En Chou:** data curation, supervision. **Fong‐Fu Chou:** validation, supervision. **Si‐Yuan Wu:** supervision, data curation. **Po‐Chih Kuo:** conceptualization, supervision, software. **Shih‐Min Yin:** conceptualization, data curation, writing – original draft, methodology, software. **Shun‐Yu Chi:** conceptualization, supervision.

## Funding

The authors have nothing to report.

## Ethics Statement

This study was approved by the Institutional Review Board of Chang Gung Medical Foundation (IRB No. 202600215B0).

## Consent

The requirement for informed consent was waived due to the retrospective nature of the study.

## Conflicts of Interest

The authors declare no conflicts of interest.

## Supporting information


**Table S1:** Missing data distribution in train set.

**Table S2:** edm270297‐sup‐0002‐TableS2.docx.

## Data Availability

The data that support the findings of this study are available on request from the corresponding author. The data are not publicly available due to privacy or ethical restrictions.
