# Supplementary material for: A Clinically Aligned Two‐Stage Machine Learning Framework for Predicting Hungry Bone Syndrome After Parathyroidectomy
Source: Endocrinol Diabetes Metab. 2026 Jul 30;9(5):e70297. doi: 10.1002/edm2.70297 (PMC13421089; doi:10.1002/edm2.70297)
Supplement: Supplementary file 1 — Table S1: Missing data distribution in train set. [file EDM2-9-e70297-s001.docx]

**Supplementary Table S1. Missing Data Distribution in train set**

| **Feature** | **Total missing count** | **%** |
| --- | --- | --- |
| Preop Neutrophil | 499 | 56.58 |
| Postoperative 1-year ALP | 447 | 50.68 |
| Postoperative 1-year P | 406 | 46.03 |
| Postoperative 1-year iPTH | 398 | 45.12 |
| Postoperative 1-year Ca | 393 | 44.56 |
| Preoperative lymphocyte | 353 | 40.02 |
| Preoperative ALP | 110 | 12.47 |
| Preoperative Bone density | 75 | 8.5 |
| Parathyroid weight (LL) | 74 | 8.39 |
| Parathyroid weight (RL) | 69 | 7.82 |
| Preoperative P | 66 | 7.48 |
| Parathyroid weight (LU) | 65 | 7.37 |
| Parathyroid weight (RU) | 55 | 6.24 |
| Preoperative Ca | 54 | 6.12 |
| Preoperative P | 54 | 6.12 |
| Parathyroid size (LL) | 49 | 5.56 |
| Parathyroid size (RL) | 40 | 4.54 |
| Parathyroid size (LU) | 39 | 4.42 |
| Parathyroid size (RU) | 37 | 4.2 |
| ESRD yrs | 22 | 2.49 |
| Preoperative Hb | 14 | 1.59 |
| OP time | 8 | 0.91 |
| BW | 7 | 0.79 |
| BMI | 7 | 0.79 |
| BW | 6 | 0.68 |
| DM | 4 | 0.45 |
| EBL | 3 | 0.34 |
| HTN | 3 | 0.34 |
| calciphylaxis | 3 | 0.34 |
| Itching | 3 | 0.34 |
| Weakness | 3 | 0.34 |
| Dialysis modality | 3 | 0.34 |
| Bone pain | 1 | 0.11 |
| Thyroidectomy status | 1 | 0.11 |
| Age | 0 | 0 |
| Postoperative iPTH | 0 | 0 |
| Sex | 0 | 0 |
| Postoperative Ca | 0 | 0 |
| Thymectomy | 0 | 0 |
| OP method | 0 | 0 |
