# Supplementary material for: A Clinically Aligned Two‐Stage Machine Learning Framework for Predicting Hungry Bone Syndrome After Parathyroidectomy
Source: Endocrinol Diabetes Metab. 2026 Jul 30;9(5):e70297. doi: 10.1002/edm2.70297 (PMC13421089; doi:10.1002/edm2.70297)
Supplement: Supplementary file 2 — Table S2: edm270297‐sup‐0002‐TableS2.docx. [file EDM2-9-e70297-s002.docx]

**Supplementary Table S2**

| **Model** | **AUROC** | **Brier** | **ECE** | **Cut-off** | **Accuracy** | **Precision** | **Recall** | **F1** | **Specificity** |
| --- | --- | --- | --- | --- | --- | --- | --- | --- | --- |
| **EasyEnsemble Two stage** | 0.776 | 0.168 | 0.052 | 0.575 | 0.757 | 0.835 | 0.815 | 0.824 | 0.623 |
| **No SMOTE** | 0.786 | 0.160 | 0.070 | 0.755 | 0.701 | 0.890 | 0.653 | 0.753 | 0.811 |
| **Single Stage** | 0.782 | 0.166 | 0.073 | 0.711 | 0.723 | 0.887 | 0.694 | 0.778 | 0.792 |
| **Preop only** | 0.769 | 0.168 | 0.073 | 0.687 | 0.718 | 0.849 | 0.726 | 0.783 | 0.698 |

The EasyEssmble model was compared to the best model under different preprocessing setting, including single stage framework, model without SMOTE for class imbalance and model using preoperative features only. Although the no-SMOTE and single-stage models yielded slightly higher AUROC values of 0.786 and 0.782, respectively, these models demonstrated substantially lower recall than the two-stage model.
